# A New Form of Tin

**Published:** 1879-01

**Authors:** 


### A NEW FORM OF TIN.

I have recently received a sample of pure tin in shred, or rather fibrous form, which is said to be an improvement over foil for filling teeth. First, in the facility with which it can be manipulated, especially in respect to construction and adaptation. Second, it is claimed that it will weld more readily and more perfectly than foil.

If the use verifies these claims the preparation is an important one. Tin is one of the important materials for filling teeth, and any one that will induce a further use of it should, and will, receive general approbation.

Further report well be made upon it after satisfactory trial.

